# COMMUNITY ENGAGEMENT IN DIFFERENT NATIONAL CONTEXTS: BUILDING BRIDGES AND AGE-FRIENDLY ENVIRONMENTS

**DOI:** 10.1093/geroni/igae098.1295

**Published:** 2024-12-31

**Authors:** Oskar Jonsson, Peggy Chi, Ian Johnson

**Affiliations:** Chair; Co-Chair; Discussant

## Abstract

To bridge the gap between research and practice, initiatives to facilitate knowledge mobilization in tandem with community engagement have become increasingly important. This symposium will advance the understanding about knowledge mobilization processes and community engagement efforts by offering insight from scholars from established research centers with long-standing community engagement as well as new initiatives connecting disparate disciplines and sectors. Representing different national contexts, the speakers will share their experiences regarding the complexities of partnership and engagement approaches, processes, and activities. Benefits (research quality, relevance, utilization, transgressing boundaries, addressing complex problems) as well as barriers (diverse conditions/cultures, lack of time/resources, multiple levels of terminology, hard-to-reach groups, projectification, boundaries of traditional science systems) will be addressed. The first speaker will describe the development and establishment of a pool of interested parties in Sweden to systematize and facilitate communication, entryways to research studies, and user involvement in aging research. The second speaker will describe the work of a center serving older African Americans from Detroit, Michigan, including innovative leadership opportunities created for its members. The third speaker will describe new initiatives in the Canadian context that purposefully choreograph knowledge mobilization activities to disrupt disciplinary and industry silos in the practice and research of salutogenic/healthy long-term care environments. The last speaker will describe an engagement effort towards age-friendly infrastructures in rural municipalities in Canada. Finally, discussant Ian Johnson from the University of Texas San Antonio will identify, compare, and discuss underlying themes, commonalities, differences, and lessons learned generated from the presentations.

